# DOCK8 in immune cells: roles and mechanisms

**DOI:** 10.3389/fimmu.2026.1854442

**Published:** 2026-06-16

**Authors:** Zixuan Liao, Sicheng Luo, Weijie Shen, Huiru Lv, Zekai Xu, Li Xu

**Affiliations:** 1 First Affiliated Hospital, Gannan Medical University, Ganzhou, Jiangxi, China; 2 Key Laboratory of Innate Immunity and Chronic Inflammatory Diseases, Jiangxi Provincial Department of Education; Key Laboratory of Prevention and Treatment of Cardiovascular and Cerebrovascular Diseases, Ministry of Education; School of Basic Medicine, Gannan Medical University, Ganzhou, China

**Keywords:** Cdc42, cytoskeleton, DOCK8, immune cells, immunodeficiency syndrome

## Abstract

DOCK8 (dedicator of cytokinesis 8) is a prominent member of the DOCK−C subfamily and functions as an atypical guanine nucleotide exchange factor (GEF). It primarily activates Rho family small GTPases—most notably Cdc42—to orchestrate cytoskeletal remodeling, and it integrates multiple key signaling pathways to preserve the functional integrity of immune cells. Recent work has established DOCK8 as a central player in both innate and adaptive immunity. In T cells, DOCK8 contributes to migration, immunological synapse assembly, lineage specification, and long−term survival, thereby supporting antiviral defense and immune homeostasis. In B cells, it fine−tunes BCR signaling, amplifies CD19−driven signals, coordinates metabolic reprogramming, and facilitates germinal center reactions, all of which underpin humoral immunity and immunological memory. In NK cells, DOCK8 influences cytotoxic activity and tissue localization by modulating immunological synapse architecture and proximal receptor signaling. In dendritic cells and macrophages, it helps regulate chemotactic migration and immune activation by precisely controlling Cdc42 activity and cytoskeletal dynamics. Mechanistically, DOCK8 serves as a signaling hub that integrates inputs from the TCR, BCR, and Toll−like receptors, coordinating the establishment of cell polarity, directed migration, immunological synapse stability, and effector function. Deficiency of DOCK8 leads to a complex immunodeficiency syndrome characterized by impaired immune surveillance, heightened susceptibility to viral, bacterial, and fungal infections, as well as allergic manifestations and disrupted immune tolerance. Although considerable progress has been made in understanding DOCK8, the integrated mechanisms operating across different immune cell types and signaling networks still await systematic elucidation. Future investigations should focus on dissecting multi−tiered signaling networks and exploring targeted interventional strategies.

## 1 Introduction

Proper function of the immune system relies on the directed migration, cell-cell interactions and dynamic execution of effector functions of immune cells within complex microenvironments. Guanine nucleotide exchange factors (GEFs) play a critical role in regulating immune cell activities (1). As a group of atypical GEFs, the dedicator of cytokinesis (DOCK) family has attracted extensive research interest in recent years. Distinct from canonical Dbl-family GEFs, DOCK proteins lack the DH-PH domain and exert their biological functions via two conserved domains, namely DOCK homology region 1 (DHR1) and DOCK homology region 2 (DHR2) (2). According to their sequence and structural features, the DOCK family is classified into four subfamilies (DOCK-A to DOCK-D) (3). DOCK8 belongs to the DOCK-C subfamily and exhibits a relatively unique expression profile in the immune system (4). DOCK8 is highly expressed in the hematopoietic system, with prominent enrichment in T cells, B cells and other immune cell populations. Functional studies have demonstrated that DOCK8 modulates the activity of small GTPases such as Cdc42 to regulate cytoskeletal remodeling, thereby governing immune cell migration, morphological maintenance and cell-cell interactions (5, 6). Accumulating evidence reveals that beyond its role in cell motility, DOCK8 also facilitates the formation and stabilization of immunological synapses and modulates multiple receptor-mediated signaling pathways. These findings indicate that DOCK8 acts not only as a cytoskeletal regulator but also as a key hub for signal integration (7).

Compared with other DOCK family members, DOCK8 possesses unique functions as a pivotal molecule for maintaining the diversity of TCR and BCR repertoires, eliminating autoreactive clones, and balancing Th2 immune responses. Genetic loss of DOCK8 causes a severe combined immunodeficiency. This disorder is characterized by restricted and aberrant TCR repertoires, defective BCR development, and robust Th2 polarization, which collectively lead to a complex clinical syndrome featuring severe infections, allergic diseases, autoimmunity and increased susceptibility to malignancies (8–10). In humans, germline defects in the *DOCK8* gene result in severe pathological phenotypes. In 2009, several research groups independently reported that *DOCK8* mutations give rise to a novel autosomal recessive combined immunodeficiency, termed DOCK8 deficiency syndrome (DIDS) (11, 12). Such a clear correlation with a distinct human disease spectrum is rare among the DOCK family members. This disease is characterized by recurrent viral, bacterial and fungal infections, accompanied by markedly elevated serum IgE levels, eosinophilia and severe allergic inflammation (12). Patients also present with impaired T cell function, defective germinal center responses and disrupted immune tolerance. These clinical manifestations are highly consistent with the functional defects of DOCK8 in diverse immune cell subsets. For instance, impaired T cell migration and immunological synapse formation account for weakened antiviral immunity. Abnormal germinal center responses and dysregulated BCR signaling in B cells lead to humoral immunodeficiency and elevated IgE. Defective development and cytotoxicity of NK and NKT cells increase susceptibility to infections and malignancies. Meanwhile, impaired Treg function and broken immune tolerance contribute to allergic and autoimmune phenotypes. The close correlation among cellular phenotypes, functional abnormalities and clinical manifestations further underscores the essential role of DOCK8 in cytoskeletal regulation and immune signal integration (11).

Although research on DOCK8 has advanced substantially in recent years and its overall roles in the immune system have been gradually elucidated, several critical scientific questions remain to be addressed. First, a systematic explanation is still lacking regarding how DOCK8 exerts cell-type-specific regulatory functions across distinct immune cell populations. Second, the precise molecular mechanisms underlying the crosstalk between DOCK8-mediated cytoskeletal remodeling and signal transduction have not been fully clarified. Furthermore, it remains poorly understood how these molecular abnormalities integrate at the organismal level to ultimately drive immunodeficiency and disease pathogenesis. Resolving these issues will deepen our understanding of immune regulatory networks and facilitate the development of targeted therapeutic strategies. Herein, we systematically review the functional characteristics, molecular mechanisms and pathological implications of DOCK8 in various immune cells.

## 2 The role of DOCK8 in innate immune cells

### 2.1 Regulation of innate lymphoid cells (ILCs) by DOCK8

DOCK8 exerts distinct subset-specific effects in innate lymphoid cells (ILCs). By regulating cytoskeletal dynamics via Cdc42 and integrating cytokine signaling, DOCK8 plays a critical role in maintaining mucosal immune homeostasis.

In humans, DOCK8 deficiency commonly leads to a marked reduction in ILC3 numbers and a mild decrease in ILC2 populations. Meanwhile, ILCs exhibit functional impairment, downregulated IL-7R expression, diminished responses to IL-7, IL-23 and IL-12 stimulation, as well as increased apoptosis. Mechanistically, loss of DOCK8 profoundly impairs IL-7-dependent STAT5, IL-23-dependent STAT3 and IL-12-dependent STAT4 phosphorylation, indicating that DOCK8 broadly regulates core signaling pathways in ILCs (13). Nevertheless, individual ILC subsets display divergent responses to DOCK8 deletion, a phenomenon that is especially prominent in mouse models and sheds light on the underlying mechanisms. Unlike human cells, mouse cells do not exhibit impaired IL-12-dependent STAT4 phosphorylation upon DOCK8 loss, nor is IL-7R expression altered in mouse ILC3s. In DOCK8-deficient mice, intestinal ILC2s undergo expansion, whereas the number of ILC3s is substantially reduced. Within the ILC2 subset, DOCK8 primarily restrains aberrant expansion and excessive activation. DOCK8 deletion leads to a marked increase in intestinal ILC2s, accompanied by elevated secretion of IL-5 and IL-13. This shift drives the immune response toward a type 2 immunity dominated by Th2-related cytokines and effector cells. The inhibitory effect of DOCK8 on ILC2 over proliferation is likely mediated by Cdc42 activation (14). In contrast to ILC2s, DOCK8 mainly sustains cell survival in ILC3s. Although DOCK8 deficiency does not interfere with the differentiation of RORγt^+^ ILC3s, it causes a drastic reduction in intestinal ILC3 numbers. Further studies have demonstrated that the loss of ILC3s arises from enhanced apoptosis and impaired survival signaling in these cells (15, 16). Mechanistically, DOCK8 preserves cytoskeletal stability via Cdc42 and sustains IL-7 and IL-23-driven signaling, including STAT3 and STAT5 phosphorylation. These events collectively support ILC3 survival and the production of its effector cytokine IL-22 (15, 16). Given the essential roles of IL-22 in maintaining intestinal barrier integrity and host defense against pathogens (17, 18), impaired ILC3 function resulting from DOCK8 deficiency may compromise mucosal immune protection (**Table 1**).

**Table 1:** The role of DOCK8 in innate immune cells.

Immune cell type	Sample type	Major phenotypes upon DOCK8 deficiency	Core molecular mechanisms/downstream pathways	Ultimate immunological consequences
ILC1	DOCK8-deficient patients	Impaired proliferation, increased apoptosis; reduced response to IL-12	STAT3 (IL-23-dependent), STAT4 (IL-12-dependent), STAT5 (IL-7-dependent), Cdc42-dependent cytoskeleton	Decreased IFN-γ-mediated innate anti-infection capacity
ILC2	Human patients; DOCK8-deficient mice	Human: mild reduction, functional impairment; Mouse: marked expansion of intestinal ILC2, elevated IL-5/IL-13		Th2 skewing, enhanced type 2 inflammation, increased allergy susceptibility
ILC3	Human patients; DOCK8-deficient mice	Significantly reduced numbers; increased apoptosis; decreased IL-22 production		Impaired mucosal barrier function, reduced antibacterial defense
NK cells	DOCK8-deficient patients	Decreased cytotoxicity; reduced IFN-γ/TNF-α secretion; abnormal immune synapse; impaired migration	DOCK8–WASP–Talin complex; Cdc42-mediated F-actin remodeling; abnormal LFA-1 polarization; impaired NKp30/Lck proximal signaling; decreased CCR7 expression	Weakened antiviral and antitumor immune surveillance
Macrophages	DOCK8-deficient mice	Reduced migration capacity; abnormal polarization; impaired phagocytosis under certain conditions	DOCK8–LRAP35a–Cdc42 axis; actin-myosin coupling; IL-10–STAT3/WASP pathway	Impaired directional migration and anti-inflammatory function
Dendritic cells	DOCK8-deficient mice	Severe migration defects in 3D environments; reduced lymph node homing; impaired antigen presentation	Spatial activation of Cdc42 at the leading edge	Decreased T cell priming efficiency, impaired adaptive immune activation
Neutrophils	DOCK8-deficient patients; S. aureus-infected mice; sepsis mouse models	Mild chemotactic defects in humans; increased death and reduced phagocytosis in skin infection; enhanced chemotaxis and inflammation in sepsis	Maintenance of survival and phagocytosis in infectious environment; in sepsis, DOCK8 downregulation promotes glycolysis and inflammatory programs	Reduced skin antibacterial defense; enhanced inflammation in sepsis
Mast cells	DOCK8-deficient mice; patient skin biopsies	Enhanced degranulation; exacerbated passive cutaneous anaphylaxis (PCA) reaction; expansion of mucosal mast cells (MMCs); increased tryptase^+^ cells in human skin	Regulation of degranulation and immune microenvironment	Enhanced allergic reactions and food allergy

A comparison between ILC2s and ILC3s reveals that DOCK8 exerts opposing regulatory effects on distinct ILC subsets in mice: it restrains proliferation in ILC2s while supporting cell survival in ILC3s. Such divergence may stem from distinct patterns of Cdc42 dependency between the two cell types. In ILC2s, Cdc42 primarily mediates signaling for cellular activation and expansion, whereas in ILC3s, it mainly maintains structural stability and anti-apoptotic capacity. Furthermore, cellular microenvironments may also determine the functional polarity of DOCK8. ILC2s are predominantly regulated by IL-33, while ILC3s rely heavily on IL-23 and IL-7.By coordinating cytoskeletal dynamics and cytokine signaling under diverse immune conditions, DOCK8 acts as a key molecule linking immune tolerance and host defense. DOCK8 deficiency not only exacerbates ILC2-mediated allergic inflammation, but also impairs ILC3-dependent mucosal barrier defense. This dual defect accounts for the concurrent phenotypes of unrestrained inflammation and heightened susceptibility to infection observed in this disease.

### 2.2 Regulation of natural killer cells by DOCK8

DOCK8 also mediates coordinated regulation of cytoskeletal structure and signaling in natural killer (NK) cells. By integrating cytoskeletal remodeling, immunological synapse formation and proximal receptor signaling, it is indispensable for sustaining the effector functions of NK cells.

Structurally, DOCK8 modulates NK cell cytotoxicity primarily by regulating immunological synapse formation. Studies have demonstrated that NK cell cytotoxicity is markedly impaired in patients with DOCK8 deficiency, and this functional defect is tightly linked to abnormal immunological synapse architecture (19, 20). In humans, DOCK8 loss does not alter the overall F-actin content in NK cells, but disrupts localized actin rearrangement at the immunological synapse. This defect impairs polarization of the integrin LFA-1 and directional trafficking of cytotoxic granules toward target cells, ultimately leading to defective lytic synapse formation (19, 20). Further evidence shows that DOCK8 forms a complex with Wiskott–Aldrich syndrome protein (WASP) and talin. Via a Cdc42-dependent mechanism, it coordinates actin remodeling and integrin-mediated adhesion to guarantee efficient execution of NK cell effector functions (20). Collectively, these findings suggest that the binding between LFA-1 and ICAM-1 facilitates the assembly of the DOCK8-Talin-WASP complex. This complex activates WASP to drive cytoskeletal remodeling, which in turn promotes the polarization of cytotoxic granules toward the immunological synapse (7). DOCK8 also acts as a vital regulator in signal transduction. Studies using NK cells from DOCK8-deficient patients revealed that engagement with target cells or activation of the NKp30 receptor suppresses downstream signaling mediated by Src-family kinases, particularly Lck. This results in a prominent reduction in the production of key cytokines including IFN-γ and TNF-α (21). Notably, stimulation with PMA and ionomycin partially restores cytokine secretion, indicating that this functional defect primarily occurs at the proximal receptor signaling stage (21). These two lines of evidence suggest that the structural and signaling defects caused by DOCK8 deficiency are mechanistically coupled. Immunological synapse formation relies on precise cytoskeletal rearrangement. Abnormal synapse architecture resulting from DOCK8 loss may impair the assembly and stability of proximal receptor signaling complexes, thereby secondarily attenuating downstream signal transduction.

In addition, DOCK8 modulates NK cell migration and tissue distribution, as validated in patients with DOCK8 deficiency. DOCK8 downregulation suppresses surface expression of CCR7 in CD56 bright NK cells via a WASP-independent pathway. This impairment hinders their homing to secondary lymphoid organs and reduces NK cell numbers in lymph nodes (22). Meanwhile, DOCK8 mutations may disrupt immune homeostasis due to compromised cytotoxic function, a mechanism that potentially contributes to excessive inflammatory phenotypes (23) (**Table 1**).

In summary, as a core molecular hub linking cytoskeletal dynamics and receptor signal transduction, DOCK8 orchestrates NK cell function through multi-layered regulation of structural remodeling, signaling transduction and migratory capacity. DOCK8 deficiency not only impairs the effector execution of NK cells, but also disrupts their tissue distribution and immune surveillance. These collective defects profoundly influence the initiation and progression of infectious diseases, malignancies and inflammatory disorders.

### 2.3 Regulation of macrophages and dendritic cells by DOCK8

DOCK8 regulates cell polarization and directional migration primarily by activating Cdc42 and coordinating cytoskeletal dynamics. Accumulating evidence has demonstrated that although DOCK8 exhibits distinct functional patterns in macrophages and dendritic cells (DCs), its core functions in these two cell types converge on the maintenance of migratory potential and the efficient execution of immune responses.

In macrophages, DOCK8 boosts migratory capacity by regulating cell polarization and cytoskeletal dynamics. Macrophages from DOCK8-deficient mice display impaired migration. Mechanistically, DOCK8 interacts with LRAP35a to form a complex that activates Cdc42. This complex further couples with the actin-myosin network to drive cell polarization and directional movement (24). In addition, DOCK8 acts synergistically with WASP to modulate the functional state of anti-inflammatory macrophages via the IL-10-dependent STAT3 signaling pathway. This indicates that beyond migration control, DOCK8 also participates in immune regulation (25). Notably, under certain pathological conditions such as excessive heme accumulation, aberrant activation of the DOCK8-Cdc42 axis conversely suppresses the migration and phagocytic activity of phagocytes (26). This finding highlights that Cdc42 activity requires tight spatiotemporal regulation. Accordingly, classifying DOCK8 simply as a pro-migratory factor may not be accurate, as this oversimplification overlooks its role in restricting cell migration under specific microenvironmental conditions (**Table 1**).

DOCK8 is also a key regulator of migration and immune priming in dendritic cells (DCs). In DOCK8-deficient mice, DCs exhibit impaired migration toward secondary lymphoid organs, which in turn compromises their antigen presentation and T cell activation (27). Notably, this function of DOCK8 relies on its DHR-2 domain, which mediates Cdc42 activation. Loss of DOCK8 does not alter the total expression level of Cdc42, but disrupts its spatially restricted activation pattern, eventually leading to migratory defects (5). This suggests that DOCK8 not only activates Cdc42, but also restricts its aberrant activation outside the cell leading edge (**Table 1**).

### 2.4 Dual regulation of neutrophils by DOCK8

Patients with DOCK8 deficiency present neutrophil chemotactic defects (28). Wilkie et al. reported that DOCK8-deficient mice have normal peripheral neutrophil counts, accompanied by compensatory upregulation of the chemokines Cxcl1, Cxcl2 and Cxcl3. Following cutaneous *Staphylococcus aureus* infection in these mice, neutrophils undergo increased cell death and display impaired phagocytosis (29). Intriguingly, DOCK8 exerts the opposite effect in mouse models of sepsis. Reduced DOCK8 expression in neutrophils enhances aerobic glycolysis, which in turn boosts cell proliferation, chemotaxis, phagocytic activity and proinflammatory cytokine release (30). Taken together, DOCK8 deficiency impairs antibacterial defense during cutaneous infection, yet relieves immune suppression and exacerbates inflammatory injury in sepsis. This paradox featuring concurrent immune deficiency and hyperactivation indicates that DOCK8 function is highly context-dependent (**Table 1**).

### 2.5 Negative regulation of mast cell homeostasis by DOCK8

IgE-mediated food allergy is a common type I hypersensitivity reaction. As major effector cells, mast cells (MCs) participate in this process via the high-affinity IgE receptor (FcϵRI) expressed on their surface (31). Accumulating evidence reveals that DOCK8 negatively regulates mast cell activation and proliferation through two distinct aspects: intrinsic mast cell function and the immune microenvironment. For one thing, DOCK8 acts as a key negative regulator of cutaneous mast cells. Under steady-state conditions, mast cells from DOCK8-deficient mice exhibit heightened degranulation capacity, leading to aggravated IgE-dependent passive cutaneous anaphylaxis (PCA). In addition, skin biopsies from DOCK8-deficient patients show an increased number of tryptase-positive cells, suggesting that DOCK8 restricts the expansion of cutaneous mast cells *in vivo* (32). The underlying molecular mechanisms remain to be fully elucidated. For another, DOCK8 indirectly suppresses the expansion of mucosal mast cells (MMCs) by modulating T cells and the intestinal immune microenvironment. In mice, DOCK8 deficiency reduces Th17 cell populations and disrupts intestinal microbiota composition. These changes elevate IL-25 production and enhance the secretion of Th2-derived IL-4. Meanwhile, DOCK8-deficient regulatory T cells (Tregs) fail to efficiently constrain intestinal IL-4 responses, ultimately forming a pro-inflammatory positive feedback loop that drives MMC expansion (33) (**Table 1**).

Overall, myeloid cells differ markedly in their dependence on DOCK8. In macrophages and dendritic cells (DCs), DOCK8 sustains directional migration and antigen presentation within complex tissue microenvironments by regulating Cdc42 activation, cell polarization and actin remodeling. In neutrophils, DOCK8 additionally modulates cell survival, phagocytosis and inflammatory metabolism. For mast cells, it predominantly restricts degranulation and preserves local immune homeostasis. These observations indicate that DOCK8 acts in a strongly context-dependent fashion. Under steady-state conditions or localized infection, it primarily supports cellular migration and tissue defense. Conversely, during persistent inflammation, metabolic stress or tissue injury, dysregulated DOCK8 may amplify inflammatory responses and trigger functional disturbances.

## 3 The role of DOCK8 in adaptive immune cells

### 3.1 Regulation of B cell receptor signaling mediated by DOCK8

DOCK8 regulates B cell functions across distinct B cell subsets and developmental stages through multiple mechanisms, including cytoskeletal remodeling, co-receptor signal amplification, immunological synapse stabilization, metabolic modulation and innate immune signaling.

Cumulative studies have demonstrated that the DOCK8-WASP axis represents the core mechanism by which DOCK8 modulates BCR signaling. This pathway primarily acts on early events including BCR clustering, cytoskeletal rearrangement, signalosome recruitment and antigen internalization. First, DOCK8 modulates WASP-related pathways to drive actin reorganization, which provides a physical foundation for BCR signal transduction (34). In B cells from DOCK8-deficient mice, both total WASP and its activated form (pWASP) are markedly reduced, alongside decreased transcription of WASP-interacting protein (WIP) (34). Another study using mouse models revealed that DOCK8 mutation impairs the regulation of actin polymerization by pWASP during BCR activation and hinders BCR cluster formation. Additionally, at the early stage of B cell activation, DOCK8 mutation restricts signalosome recruitment and reduces antigen internalization efficiency, eventually leading to attenuated proximal BCR signaling (35). Reduced BCR signaling and altered actin organization have also been observed in B cells from DOCK8-deficient patients (35). Accordingly, DOCK8 modulates BCR signaling by regulating membrane rearrangement and signal transduction following antigen recognition. In addition, secreted high mobility group box 1 (HMGB1) suppresses actin reorganization by inhibiting the MST1/DOCK8/WASP axis. This axis in turn feedback-regulates BCR clustering and signalosome recruitment, further reducing the activation sensitivity of B cells (36).

**Figure 1:**
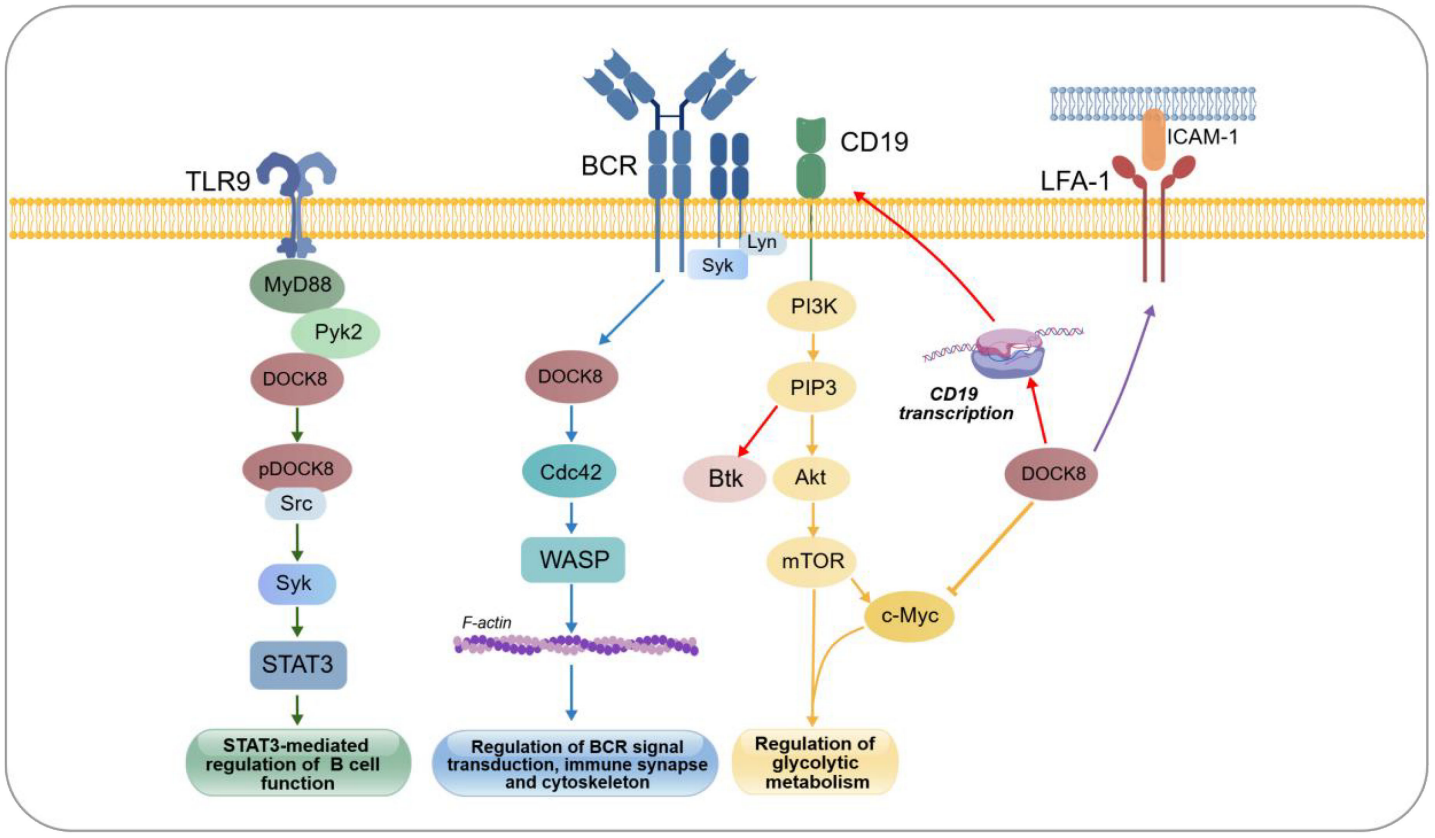
DOCK8 integrates receptor signals to coordinate cytoskeletal remodeling, metabolic reprogramming, and cell adhesion in B cells. DOCK8 acts as a core signaling hub in B cells, integrating inputs from TLR9, BCR/CD19, and adhesion receptors. Upon TLR9 activation, DOCK8 is recruited via MyD88 and Pyk2, subsequently initiating the Src–Syk–STAT3 signaling pathway (46). Meanwhile, BCR engagement activates Lyn and Syk, which promote DOCK8-mediated activation of the Cdc42–WASP–Arp2/3 axis, thereby driving actin polymerization and regulating BCR signal transduction, immune synapse formation, and cytoskeletal dynamics (34, 35). In addition, DOCK8 regulates CD19 transcription and LFA-1–ICAM-1-mediated cell adhesion, playing an important role in maintaining immune synapse stability. CD19 activates Btk and Akt in a PI3K–PIP3-dependent manner to amplify BCR signals. DOCK8 mutations downregulate the PI3K-AKT-mTOR pathway to modulate glucose metabolism, but enhance B cell glycolysis by upregulating c-Myc expression (44). Together, these pathways highlight the critical role of DOCK8 in coordinating B cell signaling, cytoskeletal remodeling, and metabolic reprogramming.

Beyond direct regulation of WASP-associated signaling, DOCK8 also modulates BCR activation and memory B cell function via the co-receptor CD19. As a critical amplifier of BCR signaling, CD19 expression directly sets the activation threshold of BCR (37). DOCK8 regulates CD19 at the transcriptional level and affects the phosphorylation of its downstream signaling molecules, including Bruton’s tyrosine kinase (Btk). B cells from DOCK8-deficient mice exhibit markedly reduced levels of phosphorylated CD19 (pCD19) and phosphorylated Btk (pBtk), indicating that DOCK8 plays a pivotal role in amplifying proximal BCR signaling (34). Studies on human B cells have demonstrated that DOCK8 deficiency disrupts signaling events during the transition from naive B cells to memory B cells, resulting in impaired memory responses in affected patients (34). WASP is known to be indispensable for memory B cell activation; it promotes this process by upregulating CD19 transcription and facilitating CD19 recruitment to the BCR complex (38) (**Figure 1**). Whether DOCK8 controls memory B cell activation through WASP remains unclarified. Evidence suggests that DOCK8 may regulate CD19 transcription via the WAVE pathway, offering a new direction for future mechanistic investigations (34).

DOCK8 exhibits more pronounced stage-specific functions during the germinal center B cell stage. Studies have shown that loss of Dock8 disrupts germinal center B cell development (34), while CD19-mediated Btk signaling has been demonstrated to control germinal center B cell formation (39, 40), suggesting that DOCK8 may participate in the germinal center reaction by regulating the CD19-Btk pathway. Recent mouse model studies further indicate that Dock8-deficient B cells can still capture and present antigens to initiate T cell activation. However, under conditions of limited antigen, the light zone checkpoint is impaired, and B cells fail to respond effectively to T cell-derived survival signals, which affects their differentiation into plasma cells and memory B cells (41). Taken together, DOCK8’s regulation of CD19-Btk signaling may not only affect proximal BCR activation (34) but also further influence the ability of germinal center B cells to integrate T cell help signals. When CD19-Btk signaling is weakened, B cells may not be able to maintain sufficient activation and adhesion, thereby reducing their competitive selection and survival advantage in the germinal center light zone.

Recent studies have found that DOCK8 regulates BCR signaling through metabolic reprogramming. The PI3K-AKT-mTOR pathway is not only essential for mitochondrial biogenesis but also involved in glucose metabolism. Studies in mice show that DOCK8 mutations downregulate PI3K-AKT-mTOR and alter glucose metabolism (42, 43). Further research has revealed that DOCK8 mutations in mice lead to metabolic reprogramming of splenic B cells, markedly increasing their glycolytic activity. Moreover, this process may be driven by upregulation of c-Myc expression (44) (**Figure 1**). Interestingly, although DOCK8-deficient mouse B cells show enhanced metabolic activity, their BCR signaling is clearly impaired (35). This suggests that the metabolic changes caused by DOCK8 deficiency do not necessarily reflect enhanced function; instead, they may represent a compensatory or dysregulated form of metabolic reprogramming. Notably, DOCK8’s metabolic regulation also exhibits clear tissue specificity. A recent study in Dock8-deficient mice found that DOCK8 is particularly important for intestinal mucosal IgA responses. Dock8 deficiency does not affect early B cell activation, migration, or IgA class switching, but it significantly impairs the maintenance of IgA-secreting plasma cells in the lamina propria of the gut. Further interactome analysis suggests that DOCK8 may interact with various metabolic and apoptosis-related proteins. IgA^+^ B cells from Dock8-deficient mice show impaired cellular respiration and an inability to properly initiate glycolysis (45). This metabolic imbalance may be functional. DOCK8’s regulation of metabolism is not fixed in direction but depends on the tissue microenvironment, differentiation stage, and cell subset in which the B cells reside.

Finally, DOCK8 integrates multiple signaling pathways to comprehensively regulate B cell function. The “Src-Syk-STAT3” pathway is critical for TLR9-driven B cell proliferation and differentiation. A study in patients with DOCK8 deficiency showed that upon activation of Toll-like receptor 9 (TLR9), DOCK8 forms a complex with the adaptor protein MyD88 and the tyrosine kinase Pyk2. DOCK8 is then phosphorylated by Pyk2, which further recruits the Src family kinase Lyn, thereby initiating the “Pyk2-Src-Syk-STAT3” cascade (46) (**Figure 1**). These findings suggest that DOCK8 participates in linking the TLR9-MyD88 pathway to downstream STAT3 signaling, acting as an adaptor-like molecule during B cell activation. That said, current evidence is largely based on observations of complex formation and downstream signaling changes; whether DOCK8 acts as a physical bridging molecule between TLR9 and STAT3 still needs further investigation (46). In addition to regulating TLR signaling, DOCK8 also plays an important role in maintaining the structure of the B cell immune synapse and ensuring effective humoral immune responses. Experiments have shown that in mice with DOCK8 mutations, B cells exhibit defective LFA-1 polarization at the immune synapse, disrupted recruitment of ICAM-1, weakened cell-cell interactions, and impaired integration of BCR signaling. These defects ultimately lead to marginal zone B cell loss, impaired germinal center responses, defective affinity maturation, and suppressed production of long-lived antibodies (7, 47). Taken together, abnormal adhesion receptor function and insufficient B-T cell interaction may be key mechanisms underlying germinal center selection failure in DOCK8 deficiency. Recent studies have also found that free heme can suppress human B cell and plasma cell differentiation by inhibiting the DOCK8/STAT3 signaling pathway (48). Meanwhile, the cytokine IL-21 can partially restore the function of regulatory B cells in the context of DOCK8 deficiency in mice (49).

In summary, DOCK8 is broadly involved in the cytokine signaling regulatory network. Rather than regulating B cell function through a single independent pathway, DOCK8 is more likely to serve as a key molecule linking cytoskeletal remodeling, BCR/CD19 signal amplification, immune synapse maintenance, and metabolic adaptation. During the early activation stage, DOCK8 promotes BCR clustering, signalosome recruitment, and antigen internalization primarily through WASP-related pathways, laying the groundwork for subsequent CD19-Btk-mediated signal amplification. During the germinal center stage, it helps maintain LFA-1 polarization and stabilize B-T cell interactions, ensuring that B cells receive sustained T cell help and promoting differentiation into plasma cells and memory B cells. Therefore, the humoral immune abnormalities caused by DOCK8 deficiency likely do not stem from impaired BCR signaling alone, but rather from a combination of insufficient early receptor activation, reduced immune synapse stability, and successive disruptions in germinal center selection. Moreover, the metabolic abnormalities observed in different tissues suggest that DOCK8’s regulation of B cell metabolism is highly context-dependent. At the same time, DOCK8 also integrates innate immune signals such as those from TLR9, further amplifying its effects on B cell proliferation, differentiation, and the maintenance of long-term humoral immunity.

### 3.2 The role of DOCK8 in T−cell migration, differentiation, and functional maintenance

**Figure 2:**
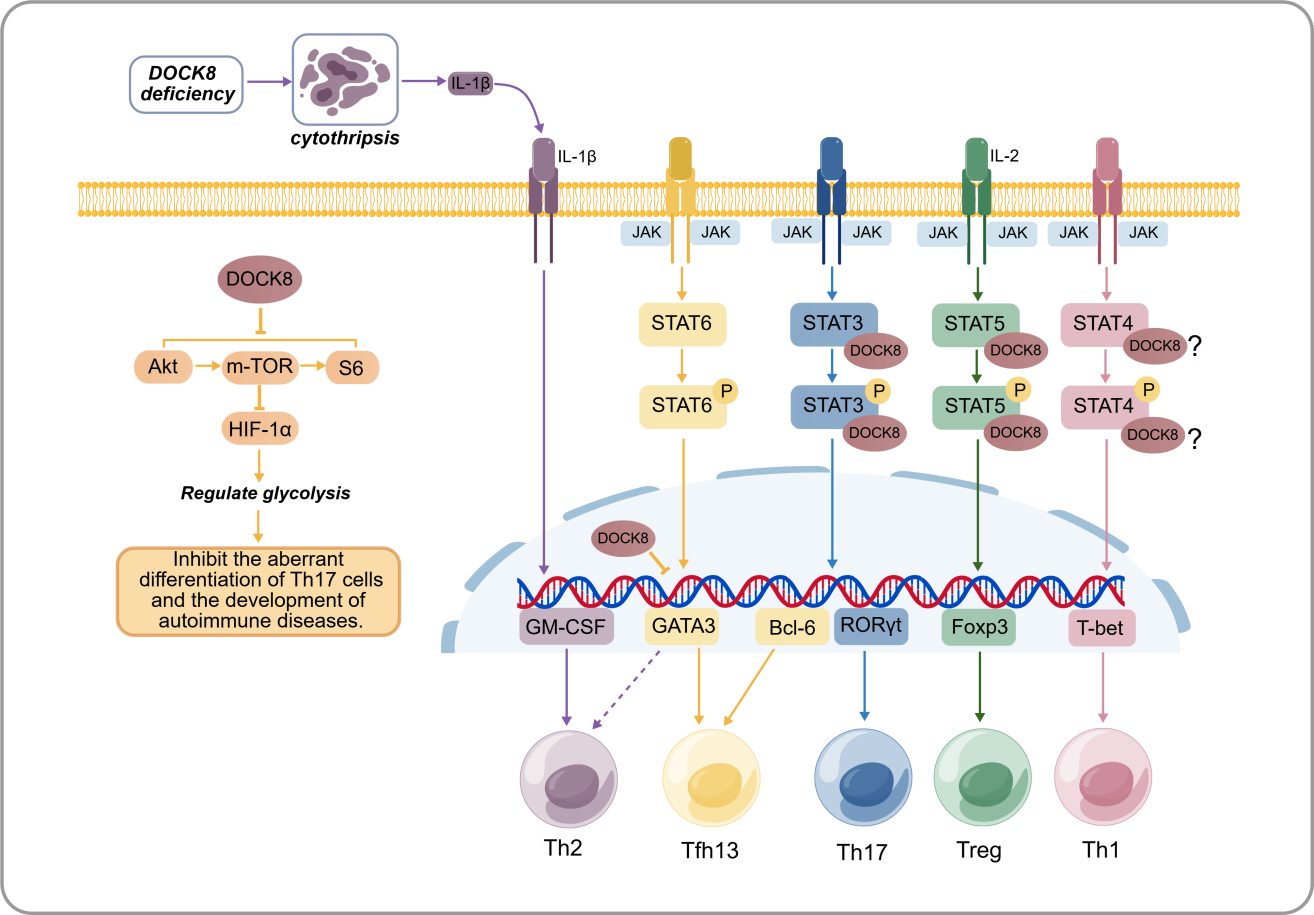
DOCK8 links cytokine signals, metabolic rewiring, and transcriptional control to guide CD4^+^ T cell differentiation. DOCK8, by coordinating cytokine signaling pathways, metabolic programs, and lineage-specific transcription factors, acts as a central molecule in regulating CD4+ T cell fate. First, DOCK8 deficiency promotes activation of the Akt–mTOR–S6–HIF-1a signaling axis and enhances glycolytic metabolism, leading to abnormal Th17 differentiation and the development of autoimmune diseases (70). DOCK8 deficiency also leads to cytothripsis, which releases inflammatory mediators such as IL-1b, subsequently inducing CD4+ T cells to produce GM-CSF and driving Th2 cell polarization (70). Cytokine signaling pathways further determine lineage commitment of the cells. DOCK8 binds to STAT3 to promote its phosphorylation and nuclear translocation, inducing RORgt expression to promote Th17 differentiation (71), while also affecting Bcl-6 expression associated with T follicular helper (Tfh)-like responses. DOCK8 suppresses GATA3 expression, thereby limiting the differentiation of Th13 cells (78). Since GATA3 is a key factor driving Th2 differentiation, when DOCK8 is deficient, this inhibitory effect may be relieved, thus promoting Th2 differentiation. At the same time, IL-2 promotes Foxp3 expression and mediates regulatory T cell (Treg) differentiation through the JAK–STAT5 signaling pathway (61). DOCK8 can interact with STAT5 and promote its phosphorylation, thereby enhancing downstream signal transduction. Since Th1 cells are influenced by T-bet mediated by STAT4, DOCK8 may also bind to STAT4 to promote its phosphorylation and nuclear translocation. In summary, these pathways together indicate that DOCK8 serves as a signaling hub that integrates extracellular signals with intracellular metabolic and transcriptional programs to determine the fate of CD4+ T cells.

DOCK8 exerts multi−tiered regulatory control over T−cell biology, with functions spanning cell migration, lineage specification, immunological synapse assembly, and the maintenance of long−term immune homeostasis. As a key upstream modulator of Cdc42, DOCK8 not only participates in cytoskeletal remodeling but also integrates cytokine, metabolic, and receptor−derived signals to influence fate decisions and functional outputs across distinct T−cell subsets (**Figure 2**). Current evidence indicates that DOCK8 deficiency does not simply reduce the numbers of a single T−cell subset; rather, it disrupts the continuous “migration-survival-differentiation-effector” regulatory axis, leading to a constellation of immune abnormalities that include heightened susceptibility to infection, exacerbated allergic inflammation, and loss of immune tolerance. In the following sections, we will review the roles of DOCK8 in T−cell biology by sequentially addressing T−cell migration, CD4^+^ T−cell differentiation and downstream subsets, CD8^+^ T−cell function, and NKT cells.

#### 3.2.1 DOCK8 regulates T-cell migration

**Figure 3:**
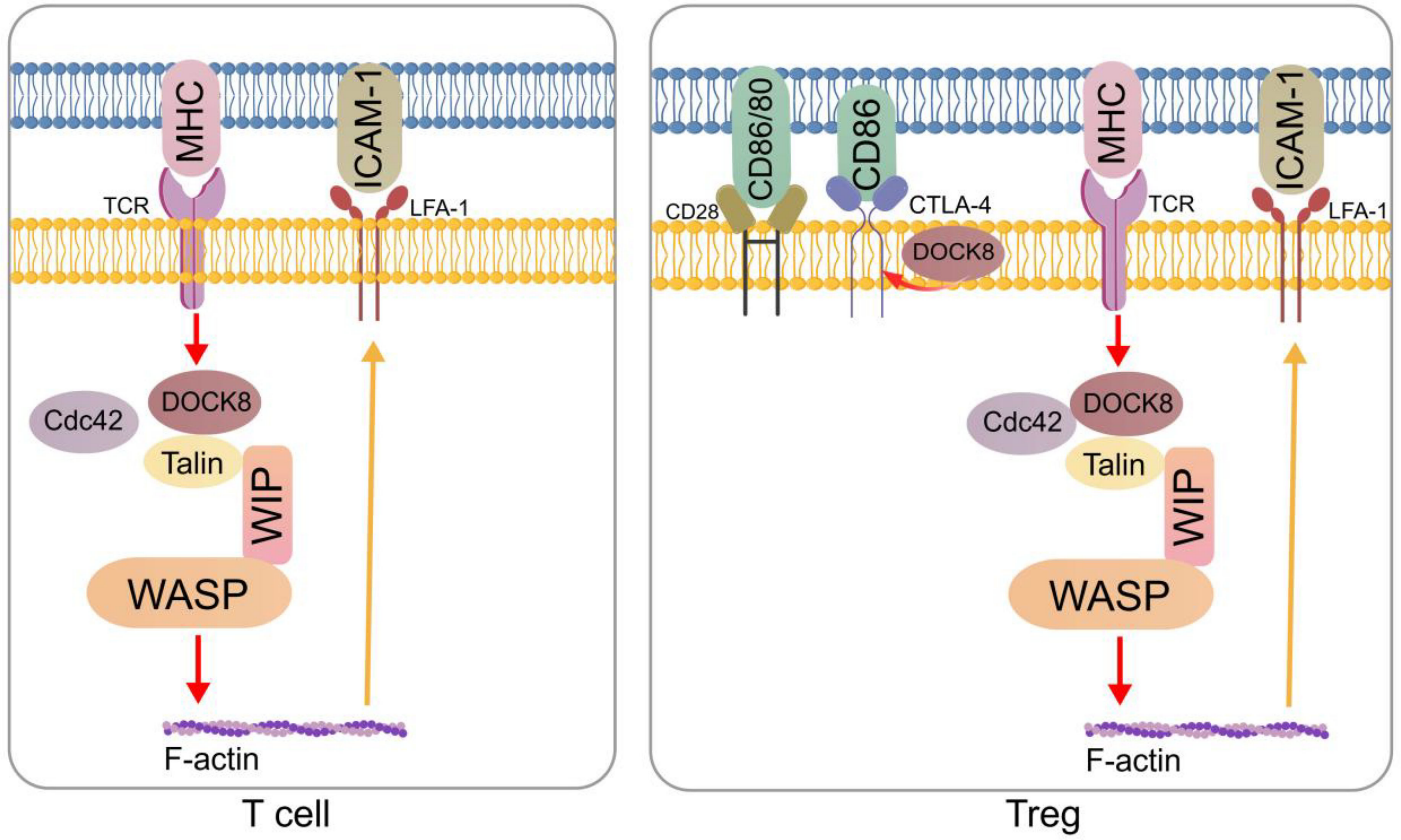
DOCK8 coordinates TCR signaling, cytoskeletal remodeling, and integrin activation to promote immunological synapse formation. Upon TCR engagement, activated DOCK8 acts as a guanine nucleotide exchange factor (GEF) to activate Cdc42. DOCK8 also serves as a scaffold molecule, linking Cdc42 to the WASP complex through interactions with WIP and Talin, thereby inducing actin cytoskeleton remodeling and F-actin polymerization. This process regulates immune synapse formation, adhesion, and recruitment of key signaling molecules. At the same time, DOCK8 controls the polarization of the integrin LFA-1 and its interaction with ICAM-1, thereby enhancing integrin-mediated cell adhesion (82). This stabilizes the immune synapse and sustains T cell activation. The bidirectional coupling between actin remodeling and integrin activation highlights DOCK8’s central role in coordinating signaling and mechanical processes during T cell responses. In Tregs, DOCK8 localizes to the F-actin ring at the immune synapse and participates in TCR-driven actin dynamics. Its loss leads to an unstable immune synapse, reduced adhesion, and impaired recruitment of key signaling molecules, which in turn weakens CTLA-4-mediated endocytosis of CD86 (60), leading to an imbalance in the CTLA-4/CD28 axis (86, 87). As a result, inhibitory signaling is reduced while co-stimulatory signaling is enhanced, leading to disrupted immune tolerance.

T cell migration is fundamental for immune surveillance, tissue homing, and inflammatory responses. DOCK8 is an important molecule in maintaining this process. In mouse model studies, DOCK8 deficiency leads to impaired thymic egress of CD4^+^ T cells. Moreover, DOCK8 maintains CD4^+^ T cell migration from the thymus to peripheral immune organs by interfering with MST-related signaling (50). Other studies have shown that in the absence of DOCK8, T cell migration in two-dimensional tissues is largely preserved, but migration in confined three-dimensional spaces such as dense tissues is significantly impaired (51). Two-dimensional migration largely reflects a cell’s intrinsic motility, whereas three-dimensional migration more closely mimics how T cells move within real tissue environments *in vivo*, requiring the cell to continuously adapt to mechanical compression, navigate through narrow spaces, and undergo rapid morphological remodeling. Thus, DOCK8’s core role is not simply to regulate “migration speed” but rather to maintain T cells’ mechanical adaptability and structural stability within complex three-dimensional tissue microenvironments. In DOCK8-deficient patients, CD8^+^ T cells often exhibit abnormal elongation, excessive nuclear deformation, and increased structural fragility during migration. Moreover, these CD8^+^ T cells undergo a non-caspase-dependent form of cell death known as “cytothripsis” (52). This indicates that DOCK8 not only affects T cell migration but also determines their survival under mechanical compression. The mechanism by which DOCK8 maintains cell stability likely involves its ability to activate Cdc42 and downstream effectors such as PAK2 to promote actin cytoskeleton remodeling, while also serving as a scaffold protein linking Cdc42 to cytoskeletal components (52). Studies in mice have shown that DOCK8 forms a signaling axis with MST1 to regulate the formation of a “central pool of F-actin” under spatially confined conditions. This structure is critical for maintaining cell morphological integrity and limiting excessive nuclear deformation. In DOCK8-deficient cells, this structure is markedly absent, leading to accumulated DNA damage, premature senescence, and reduced viability (51). Additionally, DOCK8 can form a complex with WIP and WASP to promote ARP2/3-dependent actin polymerization, participating in T cell immune synapse formation, transendothelial migration, and lymph node homing in mice (53) (**Figure 3**). Correspondingly, its activity is also finely negatively regulated; for example, LRCH1 inhibits Cdc42 activation by competitively binding to DOCK8, thereby restricting T cell migration in mice (54). Overall, DOCK8 serves as a key hub linking signal transduction, cytoskeletal dynamics, and mechanical adaptation during T cell migration.

#### 3.2.2 The effect of DOCK8 in regulatory T cells

Tregs play a central role in maintaining immune homeostasis and preventing autoimmune responses, with their development, stability, and function relying on a multi-layered molecular regulatory network (55–57). DOCK8 is critically involved in their development, survival, lineage stability, and suppressive function.

##### 3.2.2.1 DOCK8 regulates Treg development

During thymic development, DOCK8 plays a selective role in regulating Treg generation. Studies have shown that in Dock8 mutant mice, thymus-derived Foxp3^+^ Tregs and their precursors are significantly reduced, while the negative selection process remains largely unchanged. This suggests that DOCK8 is not primarily involved in TCR-mediated selection, but rather may support the survival of developing Treg cells (58). Of note, overexpression of the BCL2 gene fails to rescue the Treg reduction caused by DOCK8 deficiency, indicating that this defect does not arise from classical mitochondrial-dependent apoptosis but may involve atypical cell death mechanisms (58). These findings suggest that DOCK8’s core role during Treg development is more geared toward maintaining cell survival and developmental homeostasis, rather than directly determining Treg lineage commitment.

##### 3.2.2.2 DOCK8 regulates Treg lineage stability

In addition to affecting Treg development, DOCK8 is also involved in maintaining Treg lineage stability in the peripheral environment. Current evidence for this role comes primarily from mouse models of contact hypersensitivity (CHS). In DOCK8-deficient mice, Tregs are more prone to phenotypic reprogramming in inflammatory environments, converting into “Th1-like” Tregs that express T-bet and IFN-γ, and even further losing FOXP3 expression to become “ex-Treg” cells (59). This phenomenon indicates that DOCK8 not only determines whether Tregs can be generated, but also whether they can stably maintain their Treg identity.

##### 3.2.2.3 DOCK8 regulates Treg suppressive function and immune tolerance

Beyond development and stability, DOCK8 is also directly involved in maintaining Treg suppressive function. Mouse studies have shown that DOCK8 localizes to the F-actin ring at the Treg immune synapse and participates in TCR-driven actin dynamics. Its loss leads to an unstable immune synapse, reduced adhesion, impaired recruitment of key signaling molecules, and also affects CD86 trans-endocytosis, thereby weakening Treg-mediated suppression of antigen-presenting cells (60) (**Figure 3**). Additionally, DOCK8 maintains Treg fitness and suppressive function by regulating IL-2–STAT5 signaling in mice. IL-2 signaling is a central pathway for Treg survival and functional maintenance, and DOCK8 interacts with STAT5 and promotes its phosphorylation, thereby enhancing downstream signal transduction (60, 61). Treg-specific DOCK8 deficiency impairs STAT5 activation, which in turn affects FOXP3 expression maintenance and cellular competitive fitness, leading to impaired immunosuppressive function of Tregs (61). Of note, these two regulatory mechanisms may be intrinsically linked. Treg development and function depend on the combined maintenance of sustained TCR signaling and IL-2–STAT5 signaling (62, 63), in which TCR and co-stimulatory signals induce expression of the high-affinity IL-2 receptor α chain (CD25), and IL-2 further activates STAT5 and sustains FOXP3 transcription (**Figure 2**). DOCK8’s involvement in immune synapse and cytoskeletal remodeling may affect the stable transmission of TCR and co-stimulatory signals, thereby indirectly influencing IL-2–STAT5 pathway activation. Therefore, the impaired Treg suppressive function caused by DOCK8 deficiency is likely the result of a sequential interplay among TCR/immune synapse abnormalities, defective IL-2–STAT5 signaling, and compromised FOXP3 maintenance.

##### 3.2.2.4 Treg dysfunction caused by DOCK8 deficiency contributes to various diseases

Treg dysfunction resulting from DOCK8 deficiency is also closely linked to multiple disease phenotypes. DOCK8 deficiency-associated eczema is not merely caused by barrier defects but involves a combination of Treg dysfunction and Staphylococcus aureus colonization. In mouse models of allergic inflammation, DOCK8 deficiency leads to reduced numbers and impaired function of skin Tregs, making the host more sensitive to epicutaneous antigen sensitization (64). Furthermore, in a mouse model of contact hypersensitivity (CHS), DOCK8 deficiency exacerbates inflammation, suggesting its regulatory role in skin immune responses (59). Additionally, DOCK8 indirectly regulates B cell peripheral tolerance through its effects on Tregs, preventing the expansion of self-reactive B cell clones (65).

Overall, DOCK8 likely connects TCR signaling, cytoskeletal remodeling, and IL-2–STAT5 signal transduction. Its impact on Tregs spans multiple levels, including development, lineage stability, and immunosuppressive function, thereby jointly maintaining peripheral immune tolerance and tissue immune homeostasis. The Treg abnormalities caused by DOCK8 deficiency also suggest that Treg dysfunction may be one of the key mechanisms underlying DOCK8 deficiency-associated allergies, autoinflammation, and immune dysregulation. That said, current understanding of how DOCK8 coordinates different signaling pathways and its dynamic regulation of Treg function across various tissue microenvironments remains limited.

#### 3.2.3 DOCK8 regulates Th cell lineage differentiation: impaired Th17, Th2 skewing, and Tfh abnormalities

Beyond Tregs, DOCK8 also plays an important role in balancing helper T cell lineages. The differentiation of CD4^+^ T cells into various T helper (Th) subsets and T follicular helper (Tfh) cells is central to adaptive immune responses, and their lineage commitment is regulated by multiple factors including TCR signaling, metabolic state, cytokine milieu, and migratory behavior (66, 67). Current evidence indicates that DOCK8 deficiency does not simply impair a single Th subset but instead leads to a CD4^+^ T cell network imbalance characterized by Th2 skewing, along with Th17 dysfunction and Tfh abnormalities. Notably, this imbalance differs between humans and mice. In DOCK8-deficient patients, the main features are impaired Th1 and Th17 function along with Th2 skewing (68, 69). Whereas in mouse models, Th2 enhancement is accompanied by increased proportions and enhanced differentiation of Th1 and Th17 cells (70). These differences suggest that DOCK8 regulation of CD4^+^ T cell lineage may be species- and microenvironment-dependent.

##### 3.2.3.1 DOCK8 affects Th17 differentiation through STAT3 signaling and metabolic regulation

Studies in DOCK8-deficient patients have shown that DOCK8 deficiency inhibits Th17 cell differentiation. Mechanistically, DOCK8 interacts with STAT3 to promote its phosphorylation and nuclear translocation, thereby facilitating STAT3-dependent Th17 differentiation. When DOCK8 is absent, STAT3 activation is impaired, and the differentiation of naive CD4^+^ T cells into Th17 cells is markedly reduced (71). Since STAT3 is a key transcription factor driving Th17 differentiation in response to cytokines such as IL-6, IL-21, and IL-23, DOCK8 deficiency essentially disrupts a critical signaling axis required for maintaining Th17 lineage stability. This mechanism, primarily derived from human cell studies, is also consistent with the clinical manifestations of reduced Th17 cells and impaired mucosal antifungal immunity observed in DOCK8-deficient patients (72, 73).

On the other hand, DOCK8 is also involved in metabolic regulation of CD4^+^ T cells. Mouse model studies have shown that DOCK8 mutations aberrantly activate the Akt–mTOR–S6–HIF-1α pathway, enhancing glycolysis and oxidative phosphorylation in CD4^+^ T cells (70). Given that different Th subsets have distinct metabolic dependencies (74), this metabolic reprogramming is likely to further alter differentiation programs. Th1 and Th17 cell differentiation is regulated by mTOR signaling (75), which may be linked to the observed increase in Th1/Th17 cells in mice. Thus, the impact of DOCK8 deficiency on Th17 may be context-dependent: in humans, it tends to manifest as Th17 deficiency due to impaired STAT3 signaling, whereas in mice, metabolic abnormalities may lead to Th17 expansion (**Figure 2**).

##### 3.2.3.2 DOCK8 deficiency promotes Th2 skewing through inflammatory amplification effects

In contrast to impaired Th17 responses, DOCK8 deficiency strikingly promotes Th2 skewing, a phenotype observed in both humans and mice. In DOCK8-deficient patients, both naive and memory CD4^+^ T cells show an enhanced propensity to differentiate toward the Th2 lineage (68). The mechanisms underlying Th2 skewing caused by DOCK8 deficiency are not yet fully understood. Apart from intrinsic T cell differentiation defects, some studies suggest that tissue inflammation amplified by impaired migration may also contribute to this process. Studies in mouse models have found that DOCK8-deficient immune cells are prone to undergo cytothripsis during tissue migration, releasing inflammatory mediators such as IL-1β, which in turn induces CD4^+^ T cells to produce GM-CSF and drives Th2 polarization. Blocking IL-1β or GM-CSF abrogates this skewing (69) (**Figure 2**). Currently, the evidence for this mechanism comes mainly from mouse models, and the causal chain from “cytothripsis—release of inflammatory mediators—Th2 polarization” remains a multi-step inference. The actual contribution of this pathway in human DOCK8 deficiency still requires further validation. A recent study also found that Annexin A1 (ANXA1) enhances Th2 cell differentiation in Dock8-mutant mice, further suggesting that DOCK8-related Th2 skewing may be influenced by multiple inflammatory and metabolic regulators (76).

##### 3.2.3.3 DOCK8 regulates Tfh cell localization and functional differentiation

Follicular helper T (Tfh) cells play a central role in humoral immunity. DOCK8 regulates Tfh cells primarily through two aspects: “localization” and “differentiation.” First, DOCK8 is involved in regulating the spatial localization of Tfh cells. Mouse model studies have shown that DOCK8 deficiency does not significantly affect the generation of Tfh cells, but impairs their adhesion to B cells by inhibiting LFA-1 activation, making it difficult for them to effectively localize to the germinal center, ultimately compromising IgG antibody responses (77). Second, DOCK8 also participates in regulating Tfh functional subsets. Recent mouse studies have demonstrated that DOCK8 limits IL-13^+^ Tfh13 cell differentiation by promoting STAT3 activity and suppressing GATA3 expression; its deficiency promotes Tfh13 expansion and enhances IgE-mediated allergic responses (78) (**Figure 2**). Tfh13 cells have been recognized as an important subset driving high-affinity IgE production and allergic inflammation, and their abnormal expansion is closely associated with food allergy and severe allergic diseases (79). Therefore, DOCK8 deficiency not only leads to classical Th2 skewing but may also further amplify humoral allergic responses by promoting Tfh13 formation. Notably, there may be a potential link between DOCK8’s regulation of Tfh “localization” and “differentiation.” Stable Tfh–B cell interactions not only help maintain Tfh localization within the germinal center but may also further influence Tfh functional status and differentiation programs by continuously providing co-stimulatory signals such as TCR and ICOS signals (80, 81).

Overall, DOCK8’s regulation of CD4^+^ T cells is distinctly systematic and multi-layered. It not only participates in Treg-mediated maintenance of immune tolerance but also influences the balance among multiple effector subsets—including Th1, Th2, Th17, and Tfh—through regulation of STAT3 signaling, metabolic homeostasis, tissue migration, and the inflammatory microenvironment. The species differences observed across studies also suggest that DOCK8’s immunoregulatory effects may be highly dependent on tissue context, inflammatory state, and metabolic background. DOCK8 deficiency does not affect a single CD4^+^ T cell lineage in isolation; rather, it is more likely to cause a systemic imbalance in the CD4^+^ T cell network across multiple levels, including differentiation, migration, localization, and effector function.

#### 3.2.4 Role of DOCK8 in CD8^+^ T cell function and memory maintenance

Compared to its role in CD4^+^ T cells, DOCK8’s functions in CD8^+^ T cells are mainly reflected in survival maintenance, effector formation, and the establishment of immunological memory. Studies have shown that DOCK8 deficiency affects the number of CD8^+^ T cells and impairs their long-term antiviral capacity. First, CD8^+^ T cells from DOCK8-deficient patients exhibit significantly restricted TCR diversity, suggesting that DOCK8 plays an important role in maintaining the TCR repertoire structure and immune homeostasis (9). Second, DOCK8 regulates CD8^+^ T cells at both the primary activation stage and the subsequent memory maintenance stage, though the extent of impairment differs between these two processes. In DOCK8-deficient patients, the number of circulating naive CD8^+^ T cells is significantly reduced, and most CD8^+^ T cells display a CD45RA^+^CCR7^-^ phenotype (82). This phenotype is typically associated with exhaustion or replicative senescence (83). In Dock8-deficient mice, peripheral T cell reduction, increased turnover, and decreased survival have also been observed, suggesting a conserved role for DOCK8 in maintaining peripheral T cell homeostasis (89). During the early activation phase, DOCK8 deficiency impairs stable interactions between CD8^+^ T cells and antigen-presenting cells. Upon antigen stimulation, DOCK8-deficient CD8^+^ T cells fail to efficiently polarize the integrin LFA-1 to the immune synapse, weakening stable interactions with dendritic cells, which leads to delayed initial division and reduced activation efficiency (82)(**Figure 3**). These findings indicate that DOCK8 is involved in maintaining immune synapse stability and early activation signaling in CD8^+^ T cells. However, current evidence suggests that its impact on the primary immune response is relatively limited and not sufficient to completely block the formation of effector CD8^+^ T cells (82, 84). The impact of DOCK8 on the formation of primary CD8^+^ T cell effector responses is relatively limited.

In contrast, DOCK8 plays a more prominent role in the long-term maintenance of memory CD8^+^ T cells. Multiple studies have shown that DOCK8 deficiency does not significantly affect primary immune responses, but it markedly impairs the long-term survival and recall responses of memory CD8^+^ T cells. In mouse models of viral infection, although Dock8 deficiency allows a certain degree of initial effector response to occur, the number of memory CD8^+^ T cells gradually declines over time, and their proliferation and effector function upon re-stimulation with antigen are significantly impaired (82, 84). This observation suggests that DOCK8’s restrictive role is mainly exerted during the maintenance phase of memory CD8^+^ T cells following infection. Mechanistically, DOCK8 may support the long-term homeostasis of memory CD8^+^ T cells by maintaining cytoskeletal stability, immune synapse integrity, and sustained survival signals. Therefore, we propose that DOCK8 can be viewed as a molecule that maintains the “long-term combat capability” of CD8^+^ T cells. Its absence may not completely block the initial immune response, but it does impair memory formation and the ability to mount recall responses, ultimately reducing the body’s long-term immune defense against viruses and other pathogens. This may represent an important immunological basis for the susceptibility of DOCK8-deficient patients to chronic and recurrent viral infections.

#### 3.2.5 Role of DOCK8 in NKT cell development and functional maintenance

DOCK8 is also involved in the development and functional maintenance of NKT cells. NKT cells are originally classified as innate immune cells; however, given the similar functional mechanism of DOCK8 between NKT cells and adaptive T cells, they are discussed in the section of adaptive immunity to facilitate comparative analysis. Similar to its mode of action in other lymphocytes, DOCK8 primarily affects terminal differentiation, survival maintenance, and tissue residency in NKT cells. Studies have found that in the thymus of Dock8-deficient mice, terminally differentiated NK1.1^+^ NKT cells are significantly reduced, particularly lacking the mature NKT cell subset expressing the integrin CD103. In the liver, DOCK8-deficient NKT cells show decreased expression of the pro-survival molecule Bcl-2 and the integrin LFA-1 (85). These findings suggest that DOCK8 is not only involved in the terminal differentiation of NKT cells but also helps maintain the long-term stable presence of mature NKT cells in specific tissue microenvironments by regulating cell survival signals and integrin-dependent tissue residency. Although DOCK8-deficient NKT cells can still mount some response during the initial activation phase, their ability to sustain proliferation and produce cytokines is markedly impaired, indicating that DOCK8’s primary role likely lies in maintaining subsequent functional stability rather than determining whether they are initially activated (85). This feature is consistent with DOCK8’s role in CD8^+^ T cells and other immune cell types. Current mechanistic studies on DOCK8 regulation of NKT cells remain relatively limited. The NKT cell abnormalities caused by DOCK8 deficiency may not simply reflect a developmental defect, but rather involve the failure of mature NKT cells to receive sustained survival and functional maintenance signals within tissues.

In summary, DOCK8 plays both shared and subset-specific roles across different T cell populations. First, a number of common themes emerge across T cell subsets. These can be seen at three levels. (i) DOCK8 maintains cell migration, immune synapse stability, and tissue residency by regulating Cdc42-dependent cytoskeletal dynamics and integrin signaling, enabling T cells to migrate, survive, and interact within complex tissue environments. (ii) DOCK8 supports lineage stability and functional maintenance across T cell subsets by integrating TCR, STAT, and cytokine signals—primarily sustaining Treg-mediated immune tolerance and Th17-dependent barrier defense while restraining Th2 and Tfh13-driven allergic inflammation. (iii) DOCK8 helps maintain long-term immune homeostasis by enhancing T cell adaptation to mechanical compression, metabolic stress, and persistent antigen stimulation. Therefore, whether it is Treg instability, Th2 skewing, Tfh abnormalities, or impaired memory maintenance in CD8^+^ T cells and NKT cells, these defects can essentially be seen as different manifestations of failed long-term maintenance of cell number and function across T cell subsets in the absence of DOCK8.Second, there are still discrepancies and even apparent contradictions across studies. For example, DOCK8-deficient patients typically show reduced Th17 cells, whereas mouse models can exhibit enhanced Th17 responses. It also remains unclear whether certain effector function defects arise from cell-intrinsic abnormalities or are secondary to migration defects and tissue inflammation. These observations suggest that DOCK8’s immunoregulatory functions may be highly dependent on species background, tissue microenvironment, inflammatory state, and metabolic conditions. Future studies using conditional knockout models, single-cell omics, spatial transcriptomics, and live-cell imaging will be needed to further dissect the dynamic regulation of DOCK8 in distinct T cell states. It is precisely this combination of unifying and context-specific regulatory features that makes DOCK8 a key molecule linking cytoskeletal dynamics, signal transduction, and immune homeostasis—and also provides an important molecular basis for the complex clinical phenotypes of DOCK8 deficiency-associated immune disorders.

## 4 Current challenges and future perspectives

Despite significant progress in recent years regarding the molecular mechanisms and immune functions of DOCK8, the overall framework of its role in the immune regulatory network remains incompletely understood. Current studies indicate that DOCK8 plays important roles in both innate and adaptive immunity: it is involved in differentiation, survival, and long-term functional maintenance in Tregs, Tfh cells, Th17 cells, CD8^+^ T cells, and B cells, while also affecting migration, immune synapse formation, and effector functions in NK cells, macrophages, and dendritic cells. However, existing research has largely focused on individual cell types or isolated signaling pathways, lacking systematic integration of the shared mechanisms versus cell-type-specific functions. In particular, how DOCK8 coordinates cytoskeletal remodeling, mechanical stress adaptation, immune synapse stability, and signal integration to generate distinct functional phenotypes across different immune cells remains underexplored.

Recent studies suggest that DOCK8 is not only a cytoskeletal regulator but may also serve as a connecting molecule linking pathways such as JAK-STAT, PI3K–Akt, and NF-κB. However, the precise mechanisms remain controversial. For example, it is still unclear whether DOCK8 primarily acts through its GEF activity to activate Cdc42 or mostly functions as a scaffold protein involved in assembling WASP/WIP, MST1, and integrin complexes—and this may be highly cell-type dependent. Furthermore, whether many of the observed immune abnormalities are cell-intrinsic or secondary to migration defects and alterations in the tissue microenvironment also remains unresolved. For instance, is DOCK8-deficiency-associated Th2 skewing primarily due to intrinsic T cell differentiation defects or secondary to cytothripsis-induced inflammatory amplification? Are the germinal center B cell abnormalities and impaired CD8^+^ T cell memory maintenance direct defects or secondary manifestations of global immune dyshomeostasis? No consensus has been reached on these questions.

Currently, research on DOCK8 remains largely focused on primary immunodeficiency disorders. Its mechanisms and clinical relevance in common immune-related conditions such as inflammatory bowel disease, rheumatoid arthritis, cancer, and chronic infections remain poorly understood. Existing evidence suggests that the recurrent infections, allergy, and immune dysbalance seen in DOCK8 deficiency may not be independent of one another but rather represent the combined outcome of a continuous process involving barrier immune disruption, chronic inflammatory amplification, and loss of immune homeostasis. However, the specific mechanisms still require further validation.

Future research should focus more on key questions unique to the DOCK8 field. On one hand, it is necessary to clarify whether there is an interactive network among its roles in cytoskeletal regulation, signal transduction, and metabolic control—both in terms of common mechanisms and cell-type-specific functions. On the other hand, combining approaches such as single-cell omics, spatial transcriptomics, conditional gene knockout, and dynamic live-cell imaging will help further distinguish cell-intrinsic abnormalities from secondary effects of the tissue microenvironment, and allow deeper dissection of DOCK8’s roles in tumors, chronic infections, and persistent inflammation. At the translational level, current treatments for DOCK8-related diseases still rely mainly on hematopoietic stem cell transplantation and other replacement strategies, with a lack of precision interventions targeting the underlying molecular mechanisms. In the future, it may be possible to develop specific small-molecule regulators or targeted intervention strategies based on DOCK8’s GEF activity and its key signaling interaction networks, thereby providing a new theoretical foundation for the precise diagnosis and treatment of DOCK8-associated immune disorders.

## 5 Conclusion

In summary, DOCK8 should be understood not merely as a classical guanine nucleotide exchange factor upstream of Cdc42, but more broadly as a key molecule connecting cytoskeletal dynamics, signal transduction, metabolic adaptation, and immune homeostasis. Current evidence indicates that DOCK8 participates extensively in multiple layers of innate and adaptive immune regulation. Its roles go beyond basic processes such as immune cell migration, immune synapse formation, and tissue residency, to also influence differentiation, long-term survival, and maintenance of effector functions. Consequently, the clinical phenotype caused by DOCK8 deficiency is not a single immune defect but the combined result of migration failure, disrupted tissue homeostasis, lineage imbalance, and chronic inflammatory amplification.

Notably, different immune cells depend on DOCK8 in ways that share common features yet also show distinct differences. In NK cells, T cells, and NKT cells, DOCK8 primarily supports immune synapse stability, mechanical adaptation, and long-term survival. In CD4^+^ T cell subsets such as Tregs, Th17 cells, and Tfh cells, its role extends further to lineage stability and regulation of differentiation programs. In B cells, DOCK8 participates in BCR signal amplification, germinal center maintenance, and metabolic reprogramming. Processes such as migration, synapse stabilization, metabolic adaptation, and long-term survival likely constitute the “core common mechanisms” of the DOCK8 regulatory network, upon which different immune cells build their own specific functional phenotypes.

Moreover, there are clear species- and context-dependent differences in DOCK8-related research. For example, DOCK8-deficient patients typically show reduced Th17 cells and impaired antifungal immunity, whereas some mouse models exhibit enhanced Th17 responses and expansion of inflammatory effector T cells. It also remains unclear whether the metabolic abnormalities seen in DOCK8 deficiency are primary regulatory defects or compensatory responses to chronic cellular stress. These seemingly contradictory findings suggest that DOCK8 function is highly dependent on external factors such as tissue microenvironment, inflammatory state, metabolic conditions, and persistent antigen stimulation, rather than following a static, fixed single-pathway rule.

From a disease spectrum perspective, the recurrent infections, severe allergy, viral susceptibility, autoinflammation, and increased cancer risk associated with DOCK8 deficiency are not independent clinical phenomena but likely share a common pathological basis. On one hand, impaired function of Tregs, Th17 cells, and mucosal ILC3s disrupts skin and mucosal barrier homeostasis. On the other hand, Th2/Tfh13 skewing further amplifies IgE-mediated allergic inflammation, while migration defects and tissue damage may continuously trigger inflammatory cytokine release, leading to chronic inflammation. Therefore, DOCK8 deficiency-related diseases are better understood as a combination of barrier immune disruption, chronic inflammatory amplification, and loss of immune homeostasis. In particular, in skin and mucosal tissues, there is likely a mutually reinforcing relationship among immune cell migration defects, Staphylococcus aureus colonization, allergic inflammation, and breakdown of local immune tolerance.

At the clinical treatment level, hematopoietic stem cell transplantation (HSCT) remains the main curative option for DOCK8 deficiency. However, its efficacy is influenced by multiple factors, including transplant timing, infection burden, extent of organ damage, and residual inflammatory lesions (88). Even after successful transplantation, some patients may still have lingering chronic lung disease, persistent allergic phenotypes, or virus-related complications, suggesting that the abnormalities in tissue structure and immune microenvironment caused by DOCK8 deficiency may not be fully reversible by immune reconstitution alone. At the same time, precision interventions targeting DOCK8-related pathways also merit attention. For example, targeting the JAK-STAT, PI3K-Akt-mTOR, or cytoskeletal regulatory networks may offer new therapeutic directions for controlling inflammatory amplification and allergic responses.

In summary, DOCK8 is better understood as a key molecule that maintains the “structural stability and long-term adaptive capacity” of the immune system. The immune abnormalities caused by its deficiency are not isolated cell-intrinsic dysfunctions, but rather a systemic imbalance spanning multiple levels—including migration, tissue localization, metabolic adaptation, signal integration, and long-term homeostasis maintenance. Future studies are still needed to further dissect the dynamic regulatory rules of DOCK8 under different immune states, thereby providing a more complete theoretical foundation for mechanistic research and precision treatment of DOCK8-associated diseases.
